# Assessment of a large number of empirical plant species niche models by elicitation of knowledge from two national experts

**DOI:** 10.1002/ece3.5766

**Published:** 2019-10-25

**Authors:** Simon M. Smart, Susan G. Jarvis, Toshie Mizunuma, Cristina Herrero‐Jáuregui, Zhou Fang, Adam Butler, Jamie Alison, Mike Wilson, Robert H. Marrs

**Affiliations:** ^1^ NERC Centre for Ecology & Hydrology Lancaster UK; ^2^ Department of Botany National Museum of Nature and Science Tsukuba Japan; ^3^ Department of Ecology Complutense University of Madrid Madrid Spain; ^4^ Biomathematics & Statistics Scotland JCMB Edinburgh UK; ^5^ NERC Centre for Ecology & Hydrology Bangor UK; ^6^ School of Environmental Sciences University of Liverpool Liverpool UK

**Keywords:** biodiversity, bryophytes, forecasting, global change, species distribution model, statistical model, vascular plants

## Abstract

Quantitative models play an increasing role in exploring the impact of global change on biodiversity. To win credibility and trust, they need validating. We show how expert knowledge can be used to assess a large number of empirical species niche models constructed for the British vascular plant and bryophyte flora. Key outcomes were (a) scored assessments of each modeled species and niche axis combination, (b) guidance on models needing further development, (c) exploration of the trade‐off between presenting more complex model summaries, which could lead to more thorough validation, versus the longer time these take to evaluate, (d) quantification of the internal consistency of expert opinion based on comparison of assessment scores made on a random subset of models evaluated by both experts. Overall, the experts assessed 39% of species and niche axis combinations to be “poor” and 61% to show a degree of reliability split between “moderate” (30%), “good” (25%), and “excellent” (6%). The two experts agreed in only 43% of cases, reaching greater consensus about poorer models and disagreeing most about models rated as better by either expert. This low agreement rate suggests that a greater number of experts is required to produce reliable assessments and to more fully understand the reasons underlying lack of consensus. While area under curve (AUC) statistics showed generally very good ability of the models to predict random hold‐out samples of the data, there was no correspondence between these and the scores given by the experts and no apparent correlation between AUC and species prevalence. Crowd‐sourcing further assessments by allowing web‐based access to model fits is an obvious next step. To this end, we developed an online application for inspecting and evaluating the fit of each niche surface to its training data.

## Graphical abstract

Testing and validation is an important step in building credibility for the application of models to real‐world applications. We recruited two expert ecologists to independently evaluate the accuracy of a large number of plant species niche models for the British flora. The experts tended to agree over poorly fitting models but disagreed for models that either expert judged to be better. We make several recommendations for how the process of expert elicitation can be made more effective.

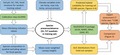

## 1 INTRODUCTION

Quantitative biodiversity models have become an important tool in our attempts to understand past ecological change and to predict what may lie ahead as humans increasingly dominate the Earth system (Ellis, 2015). The development and application of ecological models is a burgeoning field yet producing models that are credible when applied in predictive mode and easy to use is a major challenge (Evans et al., 2013; Houlahan, McKinney, Anderson, & McGill, 2017). Independent validation of the performance of models is critical if they are to win credibility and be deployed to address real problems. Recent decades have seen a rapid increase in the development and application of statistical Species Distribution or Species Niche Models (hereafter SNM) that reproduce the distributions of species based on correlative matching of presence/absence or presence‐only datasets to environmental covariates (Elith & Leathwick, 2009; Guillera‐Arroita et al., 2015). The advantage of such models is that they are easy to develop and apply. However, they have been criticized on a number of grounds. These include reliance on the assumption of niche conservatism as conditions change (Pearman, Guisan, Broennimann, & Randin, 2007), inappropriate extrapolation to future, potentially novel, configurations of environmental conditions (Yates et al., 2018), omission of demographic processes and biotic interactions (Merow et al., 2014; Zurell, Jeltsch, Dormann, & Schröder, 2009), omission of parameters linked to adaptive capacity such as phenotypic and genotypic variation and rate of likely evolution (Catullo, Ferrier, & Hoffmann, 2015). Building models that address these criticisms is essential but remains heavily data constrained given the number of species of interest. Moreover, there is no guarantee of an improvement in accuracy even if models are trained on demographic data that ought to confer realistic dynamism (Crone et al., 2011 but see Chapman, Haynes, Beal, Essl, & Bullock, 2014; Merow et al., 2014). Therefore, empirical SNM are likely to see continued development and use but in parallel with building more sophisticated hybrid models. Wise application of SNM is also fostered by the guidance emerging from a growing number of large scale tests of model transferability in space and time (Dobrowski et al., 2011; Norberg et al., 2019; Pearman et al., 2008; Yates et al., 2018).

The urgency of the problems typically addressed by SNM has also meant an increase in the formal inclusion of expert knowledge in model‐building (Addison et al., 2013; Low Choy, O'Leary, & Mengersen, 2009; Shirk, Wallin, Cushman, Rice, & Warheit, 2010) and testing (Drew & Perera, 2012; van Zonneveld, Castañeda, Scheldeman, Etten, & Damme, 2014). Confidence in the use of SNM should increase if there is a degree of consensus between model predictions and independent expert judgment. Using statistical models of the realized niche of vascular plants and bryophytes in Britain, we investigated how expert opinion can be used to rapidly evaluate a large number of SNM that have been developed for a significant fraction of the British flora, covering all common dominant and numerous rare and subordinate species. The models are freely available within an R package called MultiMOVE (Henrys et al., 2015). It is more likely that these models will be used and gain credibility if they can be shown to reproduce the response of each plant species to major ecological gradients reliably. This can be done quantitatively, by testing the ability of each model to reproduce random samples of the training data, but also by seeking the view of experts not involved in model construction but who possess comprehensive knowledge of the British flora. In this paper, we apply and compare the results of both approaches.

**Figure 1:**
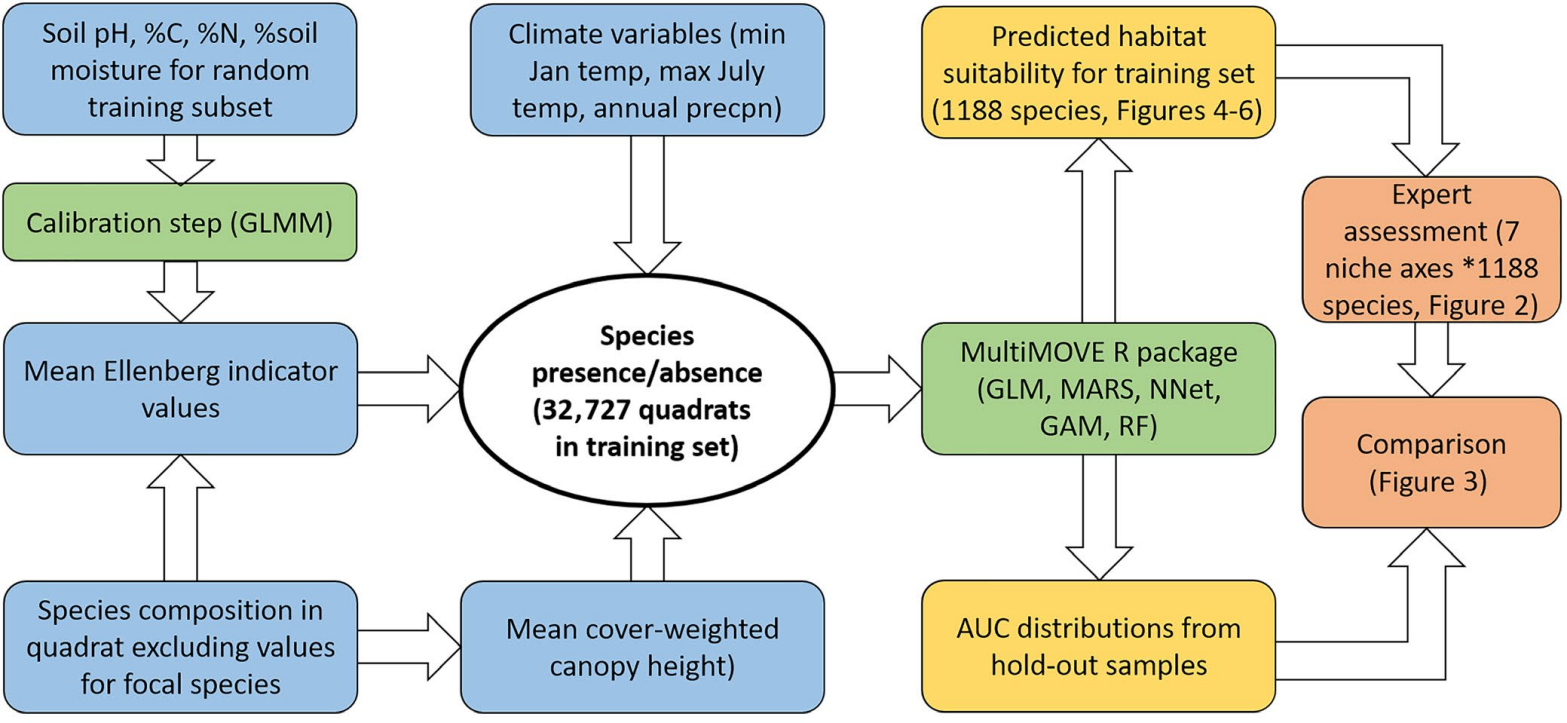
Steps involved in building and assessment of the MultiMOVE species niche models based on expert judgment and comparison with AUC. Color codes are as follows: Blue = model inputs. Green = quantitative modeling steps. Orange = Model outputs. Light red = model assessment steps. See Henrys et al. (2015) and Smart, Scott, et al. (2010) for detailed accounts of the construction of the species niche models including descriptions of the input data

Each SNM in the MultiMOVE package is a statistical representation of the realized niche of each species across British ecosystems. That is, each niche is a modeled probability space defined by the main effects and interactions between climate, vegetation height, indicators of substrate pH, fertility, and substrate wetness across the time interval in which the model‐building data were collected. A large database of species presence–absence data from quadrat locations across Britain was used to build models for 1,188 vascular plants and bryophytes (Figure 1). The availability of fine‐resolution co‐located soil measurements lends the models potentially greater accuracy in defining each realized niche (Coudun, Gegout, Piedallu, & Rameau, 2006; Wamelink, Goedhart, & Frissel, 2014) while also allowing models to be used to explore scenarios of environmental change that drive change in soil variables (Smart, Henrys, et al., 2010; de Vries, 2010). Species presence/absence data used to build the models were available at relatively fine resolution (maximum 200 m^2^ [14.14 × 14.14 m] to minimum 4 m^2^). This lessens the chance of poor model fit resulting from the averaging of environmental heterogeneity (Huston, 1999). SNM were derived by fitting species presence and absence to the explanatory variables using five different statistical modeling techniques (Figure 1). While the model development process is rigorous and scientific, in as much as it is clearly documented and therefore repeatable, it is not given that each model represents the true realized niche of each species. For example, a model may be missing important predictors, there may be insufficient occurrences to parameterize the model, or the data may not fit the assumptions of the model. To address these issues, an ensemble of modeling techniques was used recognizing that there is no single best statistical approach to species niche modeling (Araújo & New, 2006; Norberg et al., 2019; Smart, Henrys, et al., 2010). Moreover, the notion that it is possible to define the “true” realized niche as a spatially and temporally invariant pattern is problematic even though the concept of the niche remains extremely useful (Araújo & Guisan, 2006; Chase & Liebold, 2003; Pulliam, 2000). We assume pragmatically that the shape of each species’ niche is stable enough to be usefully approximated by popular niche modeling methods and, as we explore here, embodied in the experiential knowledge that can be elicited from experts (Drew & Perera, 2012; O'Hagan et al., 2006). Many of the species that we modeled have ranges that extend into the European mainland. Restrictions on data availability resulted in models that only included presence/absence for Britain thereby constraining the environmental range of some of the models to a subset of their occupied area (c.f. McCune, 2016; Thuiller, Brotons, Araújo, & Lavorel, 2004; Yates et al., 2018). A useful consequence is that we did not require experts to demonstrate knowledge of the ecological preferences of species outside Britain.

We report the results of a model assessment exercise carried out by two independent expert botanists covering all niche axes of all species in the MultiMOVE R package (Figure 1). Both experts were deemed sufficiently familiar with the habitat preferences of the British flora to be able to judge the quality of each species' model as a representation of its realized niche. Our aim was ultimately to generate species‐specific guidance for users, alerting them to potentially good and bad representations of the realized niche of each species and to help identify models in need of improvement. Clearly, the experiential impression of each niche can differ between experts depending upon the geographic and ecological scope of their familiarity with British vegetation. In this respect, two experts are better than one but not as good as an even greater number. We return to this issue in the discussion in light of an analysis of the consistency between the two experts in their assessment results for a random 5% subsample of the vascular plant species models.

Each species assessment can be broken down into three linked questions: (1) Do the response curves resulting from each of the five modeling techniques reproduce the expected niche response of the species according to the experience of the expert? (2) Since each model is fitted to a dataset of presences and absences does each model accurately predict the observations that were used to build the model? (3) Does the observed presence/absence data adequately represent the ecological range of the species in Britain? A poor representation of the niche could for example arise from biased or unrepresentative model‐building data despite the model being a good fit to these data. Since a total of 1,188 species models needed to be assessed we asked each expert to inspect the modeled response to each abiotic niche axis averaged across model types rather than evaluating each of the model types along each niche axis. Thus our principal objective was to address question 1 via an inspection of the ability of each of the ensemble models to represent the realized niche averaged across the five modeling techniques (Figure 1). We then address question 2 by generating area under curve (AUC) statistics describing the fit of each model to random hold‐out samples of the training data. The correspondence between the experts' evaluations and the model fit statistics were then compared with the expectation that better fitting models should coincide with higher expert scores for the species and niche axis combinations making up each model (Figure 1). In light of these results, we discuss the trade‐off between the time required to evaluate more complex graphical representations of model fit versus the possibility that more information‐rich visualisations could yield more accurate and comprehensive validation.

In summary, we sought to answer the following questions:

1. How did the two experts rate the ability of the models to capture the niche of each species?
2. To what extent did the experts agree with each other based on joint validation of a random subsample of the vascular plant models?
3. Did modeled species and niche axis combinations judged to be better representations of the species’ niche coincide with higher quantitative model fit statistics for each species model?

## 2 METHODS

### 2.1 Selection of experts

We circulated a request for experts to colleagues within the vegetation surveying community in Britain. Two experts were selected both of whom were prepared to commit themselves to the large size of the validation task. While we can assume that a greater number of experts should lead to more robust consensus (Drew & Perera, 2012), our investigation was limited by the funding available to pay each expert for the large number of assessments required. A previous expert‐based assessment of the habitat affinities of a subset of British plant species successfully employed three experts, hence we had no prior reason to expect that just two experts with comprehensive knowledge of the British flora would be insufficient (McInnes et al., 2017). However, in order to further identify the strengths and weaknesses of this approach we carried out a literature review of papers documenting the use of expert knowledge in validating statistical species distribution or niche models (Appendix S1). We were especially interested in the range of variation in the ratio of experts to numbers of species and in conclusions as to the usefulness of expert assessment given the levels of agreement found between experts and between experts and models.

The two expert botanists satisfied the six criteria for selection of experts in elicitation studies listed by O'Hagan et al. (2006), (a) Tangible evidence of expertise, (b) Reputation, (c) Availability and willingness to participate, (d) Understanding of the problem area, (e) Impartiality, (f) Lack of an economic or personal stake in the findings. Neither of the experts were previously acquainted with the authors either in a personal or professional capacity. Both agreed to take part in the assessment exercise and in doing so felt that their levels of botanical experience were sufficient to tackle the national scope of the assessment. Their expertise and experience of the British flora is summarized below:

*Expert 1*: This expert trained as a botanist and vegetation ecologist gaining a master degree in ecology and then further plant identification qualifications from the British Natural History Museum. The expert has 15 years' experience practicing as a professional botanist and, in the last 8 years as a professional bryologist. The expert has been a vice‐county recorder for the Botanical Society of Britain and Ireland (BSBI) for the past 12 years and a regional recorder for the British Bryological Society for 8 years.
*Expert 2*: This expert is a vegetation ecologist, bryologist and botanist with over 20 years' experience in the nature conservation sector. The expert specializes in detailed vegetation surveys especially the UK National Vegetation Classification, designing & implementing vegetation monitoring programs, training in identification and survey skills, bryophyte surveys and statistical analysis of ecological data.

In this instance, the two experts are not considered to be human research subjects in the sense of the Declaration of Helsinki and so it was not deemed necessary to seek approval and review by an Institutional Ethics Committee.

### 2.2 Assessment methodology

The modeled responses of each species along each of the seven niche axes were made available to each expert as a “shiny” application (Chang, Cheng, Allaire, Xie, & McPherson, 2016) allowing each species to be selected by the expert for inspection and scoring via a user‐friendly interface (see Figure S2.1 in Appendix S3). The modeled response curve for each niche axis was plotted as the average of the predictions generated from the GLM, GAM, MARS, and Neural Network models for the species. The Random Forest models were excluded because of the frequent occurrence of abrupt spikes in the modeled curves that were uninterpretable and probably reflected local over‐fitting (Wenger & Olden, 2012). The resource constraints of the project meant that only one average curve was plotted per niche axis rather than separate curves for each method with uncertainty intervals on each. Had we done so this would have increased the number of required assessments fourfold from 8,316 to 33,264 (1,188 species * 7 niche axes * 4 model methods) and confronted the expert with a more complex representation of each niche that would have needed longer to evaluate. We return to this issue in the discussion. The modeled response curves were derived by solving each model for values of the respective predictor. The range of the predictor variable on each *x*‐axis was defined by the maximum and minimum values in the complete training dataset used to build the models and was therefore the same for every species assessed (Henrys et al., 2015). Since each niche model included terms to be solved for other predictors these also needed to contribute to the solution of each model along each ecological gradient. This was done by setting the value of all other predictors to their median value in the training data, the default option in MultiMOVE. Hence, when inspecting a species response along a single gradient, model predictions were generated by varying the input values for this gradient only and fixing the input value for all other covariates at the median of each covariate across the training data. An alternative approach is to set the values of the background predictors to their observed values in each of the sampled locations in the training data. We explore this option later in the paper. Raw probabilities from each species' model were rescaled to account for varying prevalence in the model‐building data with the result that all values ranged between 0 and 1 (Real, Barbosa, & Vargas, 2006).

The experts were introduced to the use and installation of the software and the assessment methodology via email and telephone. A guidance note on carrying out the assessment was also circulated (see Appendix S1). Bryophyte species (*n* = 307) were assigned to one of the experts who had particular experience of the British bryophyte flora. The vascular plants (*n* = 881) were split between the two experts at random. From this pool, 45 vascular plants (5% of the total) were selected at random to be assessed by both experts. These were included among the larger list given to each expert so that neither expert knew the identity of the species that would also be inspected by the other. Experts were asked to assess the accuracy of each niche axis using four categories; poor, moderate, good, excellent (Appendix S1). No attempt was made to define this scale hence assessment was left entirely to the judgment of the expert. The exact quote from the guidance note issued to each expert is as follows:

[The niche of each species is described in terms of seven environmental axes that are all shown together on each species page;] …..[You should evaluate each of these separately by comparing what the response curve implies about the species’ preference with your experience of the species in British habitats. If unsure because you cannot understand the response or you suspect you do not have enough experience of the species' preferences throughout its range then don't hesitate to select ‘Cannot evaluate’].

### 2.3 Analysis

The results of the validation exercise are presented showing the frequency of species assigned to each class. The results for niche axes and species combinations that were assessed independently by both experts are presented as a confusion matrix showing the number of times the experts agreed and the frequency of disagreements by pairs of score; for example, by indicating how often expert 1 gave an assessment of “good” when expert 2 gave an assessment of “poor.” From these data % agreement was calculated as follows:

%agreement=(totalnumberofidenticalassessments/totalnumberofassessments)∗100.

By restricting the two sums above to just pairs containing one of the assessment categories, agreement values can also be readily calculated for each, showing for example whether experts were more likely to disagree when applying the “excellent” score or the “poor” score.

### 2.4 Comparison with quantitative model fit statistics

Area under the receiver‐operator curve (AUC) statistics for each species and each model type in the MultiMOVE ensemble were computed as follows: The presence absence data for each modeled species were split randomly into a 75% training and 25% test set. For each species and modeling method we train on the training set and predict the probability of presence on the test set. From this we calculated AUC values on the test set using the “evaluate” function in the R package dismo (Hijmans, Phillips, Leathwick, & Elith, 2011). For each species and modeling method we repeated this process 10 times and extracted the average of the AUC values. Scatter plots and a loess smoother were used to explore whether the assessment category awarded by each expert to each species × niche axis combination varied systematically with the mean AUC of the respective species model. We would for example, expect models that best predicted a hold‐out sample of their observations to be a better description of their niche and to attract a better assessment. This assumes that the observations used to build the model are representative of the species ecological range as perceived by each expert. Prevalence was plotted against mean AUC because the high true negative rates associated with species that rarely occur in the data would be expected to result in higher AUC values (Lobo, Jiménez‐Valverde, & Real, 2007; Peterson, Papeş, & Soberón, 2008). The area under curve (AUC) statistic is simply the area beneath the ROC curve, and provides a single value that is used to summarize overall performance (Boria & Blois, 2018; McCune, 2016; Yates et al., 2018).

## 3 RESULTS AND DISCUSSION

### 3.1 Expert assessment results

**Figure 2:**
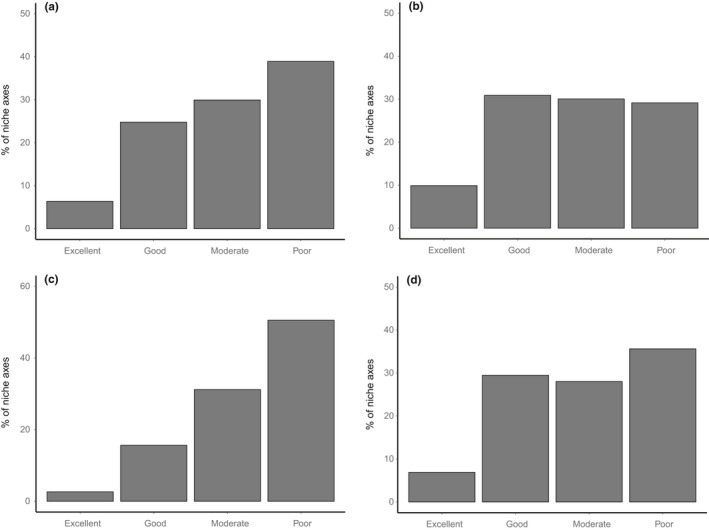
Results from assessments of the MulitMOVE models by two independent experts: (a) both experts combined. (b) Expert 1, vascular plants only. (c) Expert 2, vascular plants only. (d) Expert 1, bryophytes only

Overall, the experts assessed 39% of niche axes to be “poor” and 61% to show a degree of reliability split between “moderate” (30%), “good” (25%), and “excellent” (6%) (Figure 2a). The two experts exhibited differing tendencies in their approach to model assessment. Expert 1 assigned a greater proportion of models to categories associated with stronger model performance (Figure 2b). Expert 2 showed the reverse tendency, in particular assigning a much greater proportion of modeled niche axes to the “poor” category (Figure 2c). Since species were allocated randomly these differences cannot be attributed to any prior ecological bias in the species assessed. Expert 1 was the only expert to assess the bryophyte models. The distribution of scores was similar to results for vascular plants; 36% of model axes being considered “poor,” 28% “moderate,” 29% “good,” and 7% “excellent” (Figure 2d).

Joint assessment of a 5% random subset of vascular plant models yielded 43% agreement between experts. They were more likely to agree on the assessment of poor niche axes with increasingly less consensus about niche axes considered to be better by at least one of the experts (Table 1). These levels of disagreement are interesting; in 14 cases expert 2 assigned “poor” where expert 1 assigned “good” and in five cases expert 1 assigned “poor” where expert 2 gave “good” consistent with the tendency for expert 2 to judge more harshly than expert 1. In nine cases, disagreements centered on climate axes, in seven cases on the succession/disturbance axis conveyed by vegetation height and in the remaining 3 cases on abiotic substrate conditions. Species‐specific examples of model fits are discussed below. Model assessment scores for all species and niche axes are available in Appendix S4.

**Table 1:** Confusion matrix of results for species assessed by both experts

Expert 2	Expert 1				
	Excellent	Good	Moderate	Poor	Expert 2 totals
Excellent	2 (8)	2	1	1	6
Good	9	16 (17)	7	5	37
Moderate	9	39	44 (25)	14	106
Poor	1	14	62	64 (40)	141
Expert 1 totals	21	71	114	84	126 (43)

Note

Numbers refer to the count of niche axes and species combinations that were assessed. Thus the diagonal gives the number of assessments where both experts agreed. The figure in brackets is the % agreement for each category of score.

### 3.2 Quantitative assessment of model fit

**Figure 3:**
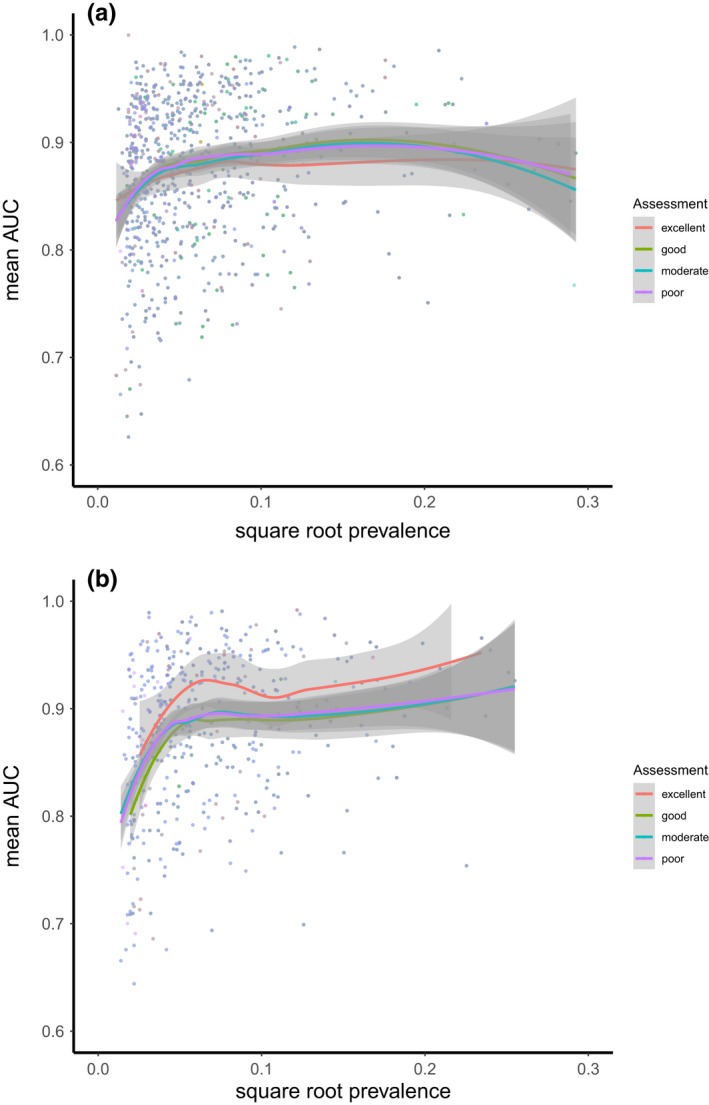
Comparison of expert assessments—(a) Expert 1. (b) Expert 2—for each species niche axis combination versus AUC statistics for the associated model and the prevalence of each species in the training data used to build each model. Loess smoothers are fitted to each species*niche axis combination grouped by the assessment category awarded by the expert. Thus each point is a species * niche axis combination whose position is defined by its prevalence on the x‐axis and the mean AUC for the species model on the *y*‐axis. Note that prevalence (the proportion of presences/ total number of quadrats) was square‐root‐transformed to spread the data more evenly across the *x*‐axis

Mean AUC statistics for the species models were invariably greater than 0.8 with most species having scores >0.9 suggesting good and excellent ability to predict the test data, respectively (Figure 3; Swets, 1988). Since a high proportion of absences is expected to decrease the false‐positive rate thereby increasing AUC, we would expect a negative correlation between species prevalence and AUC. Interestingly, while this effect cannot be ruled out, mean AUC was in fact lowest at the very lowest levels of prevalence. Regardless of the relationship between AUC and prevalence, there was no obvious difference in AUC between assessment categories for either expert (Figure 3). There was a weak indication that species models with higher AUC were more likely to be assigned as “excellent” by expert 2. However, the smoothed lines did not differ by any meaningful amount (Figure 3b).

### 3.3 Assessment results in light of the literature review

We located 25 published papers that reported an independent assessment of statistical species distribution models using expert opinion (Appendix S1). Compared to these papers, our assessment involved by far the lowest ratio of experts to study organisms (1–307 for bryophytes and 1–881 for vascular plants with 45 species evaluated by both experts). It would however, be wrong to assume that these low ratios are an accurate measure of the fraction of knowledge that could be applied by each expert to each species in the assessment. The experts were chosen based on their experience and expertise in surveying British plant communities. As such, this experience ought to have enabled assessment of the habitat preferences of each of the species embedded within the mixed‐species assemblages widely encountered by the experts. We also encouraged the experts to select the “cannot evaluate” category if they felt unable to evaluate a model through lack of experience. Even so, the levels of disagreement between the experts suggest that various unquantified biases may have influenced their judgment. For example, a species whose abiotic niche varies geographically will be wrongly evaluated if the expert's home‐range did not include the full range of the species (Drew & Perera, 2012; Murray et al., 2009; Appendix S1). In addition to these expert‐centered sources of variation, we suspect that the simplicity of the univariate model summaries may have also mitigated against more accurate (nearer to the truth) and more precise (less uncertainty surrounding estimates of the truth) assessments.

### 3.4 Trade‐offs between simple versus complex model summaries

**Figure 4:**
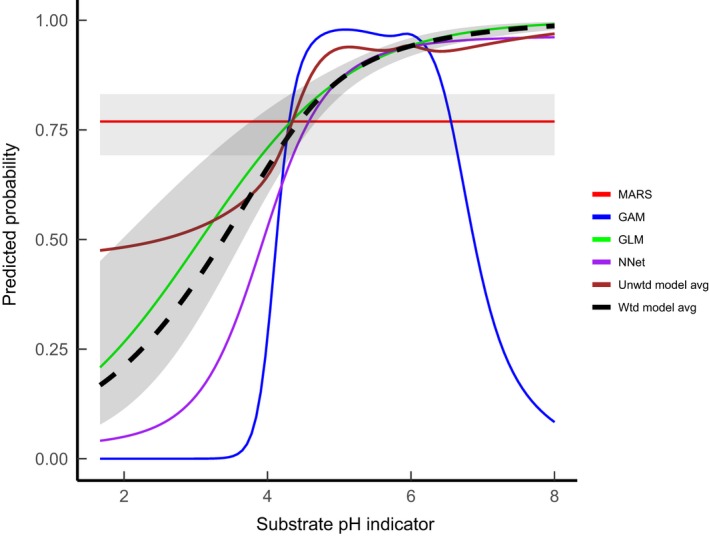
Modeled response of *Coeloglossum viride* to an indirect indicator of substrate pH. The modeled response was assessed by both experts as moderate (expert 1) and poor (expert 2). Their assessment would have been based solely on inspection of the unweighted model average (brown line). Raw probabilities have been rescaled to between 0 and 1. Gray ribbons indicate the 95% confidence region for the relevant modeled response

At least three factors come into play when evaluating each model; (a) the effectiveness of the way model fit was summarized for the expert, (b) the extent to which each model reproduces the observations used to build the model, (c) the extent to which the observational data adequately represents the ecological preferences of the species. The AUC statistics address the second issue. Across the prevalence range, mean AUC values indicated generally very good fits between the model predictions and hold‐out samples of the training data. We might therefore have expected fewer “poor” and “moderate” expert assessment scores. The two experts were able to validate the fit of each species model to each abiotic axis based on a plot of the simple model average for the five model types across each separate niche axis. Raw predicted probabilities were also standardized to range between 0 and 1 thereby allowing species to be compared on an equal basis (Figure S1.1 in Appendix S2). This simple presentation was designed to make the assessment as quick as possible. More realistic yet complex presentations are however possible, including graphing outputs from all available model types with attached confidence intervals rather than presenting just the average prediction. Expert assessors may have responded differently to such treatments but their complexity may well have meant prohibitively greater time spent on each assessment and additional training to help interpret more complex graphs. For example *Coeloglossum viride*, an orchid of shortly grazed calcareous grassland with an expected optimum at high pH and short vegetation height, was assessed by both experts. Plotting the predictions from each type of model shows how the average prediction combines outputs consistent with expectation versus models that completely fail to reproduce the expected ecological response (Figure 4). The inspection of the full range of models on the same graph would have allowed assessment and scoring of each model type as well as each axis however this will have meant a longer assessment process requiring significantly greater resourcing and training.

**Figure 5:**
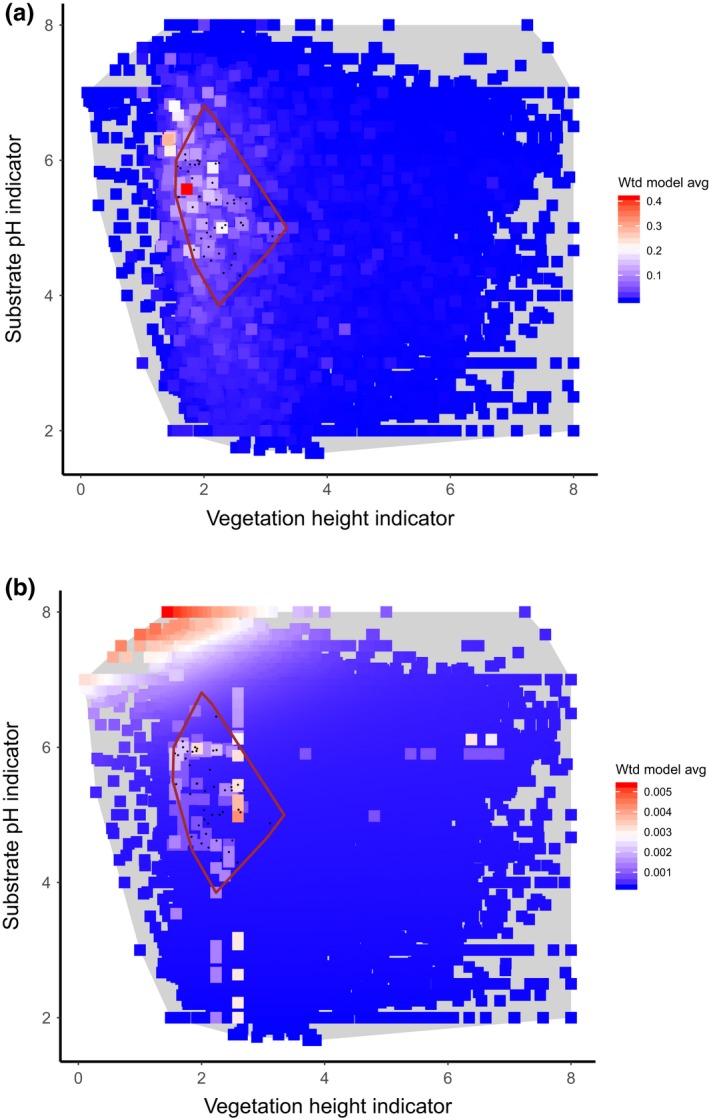
Modeled response of *Coeloglossum viride* to vegetation height (1, <10 cm, 8 ≥ 15 m), (assessed as poor by both experts) and an indirect indicator of substrate pH (assessed as moderate and poor by the two experts). Colors indicate the weighted average model prediction for all training plots in the MultiMOVE database. The red line encloses all observed occurrences of the species (black dots) in the training data. The gray polygon encloses the ecological space defined by the training data. (a) Model predictions based on observed values of background explanatory variables in each training plot. (b) Background explanatory variables set to their median values in the training data

Further insight into the way each species model represents the realized niche can be gained from examining observed data and modeled occurrence simultaneously along more than one niche axis. Such plots are better able to reveal peaks in the probability of occurrence that are not visible when predictions are averaged for all other possible axes. For example the modeled maximum probability of occurrence for *C. viride* increases when the joint response to substrate pH and vegetation height is plotted (Figure 5a). The result is a more accurate depiction of the modeled response for *C. viride* because its optimum is approximated more clearly by two rather than one niche axis (Figure 5a). The 2D plot highlights the dependence of the species on both pH and vegetation height, responses that are averaged out by examining only one dimension. However, had we presented these plots to the experts for every pair of axes this would have increased the volume of assessment material from seven graphs to 21 graphs per species.

### 3.5 The critical importance of the background variables

**Figure 6:**
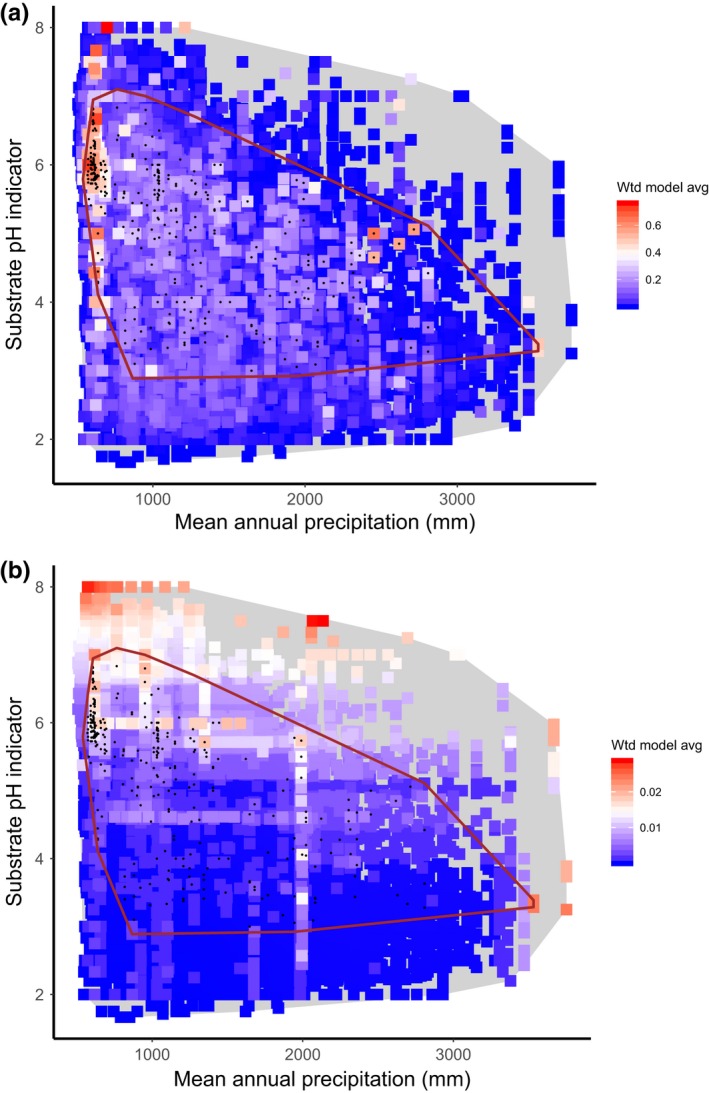
Modeled response of *Schoenus nigricans* to precipitation (assessed as good) and an indirect indicator of substrate pH (assessed as moderate). Colors indicate the weighted average model prediction for all training plots in the MultiMOVE database. The red line encloses all observed occurrences of the species (black dots) in the training data. The gray polygon encloses the ecological space defined by the training data. (a) Predictions based on observed values of background explanatory variables in each training plot. (b) Background explanatory variables set to their median values in the training data

Another important difference in the way model responses can be summarized centers on the choice of values for background variables; that is those explanatory variables other than the ones that define the particular abiotic gradient being assessed. The default setting in MultiMOVE is to set the background variables to the median for the input data. This effectively holds all other variables constant allowing predictions to vary only in response to the gradient of interest. However, the assessment results show that this can lead to predictions being made for unrealistic combinations of explanatory variables while at the same time missing those conditions that are optimal with respect to the observed occurrences of the species. Turning again to *C. viride*, when all explanatory variables other than pH and vegetation height are set to the median values for the training data unrealistic predictions are generated outside of the observed range of the species. Moreover all predicted habitat suitability values are extremely low (Figure 5b). Predicting across the same two gradients but solving the model based on observed values at each sampled location for all other explanatory variables results in the region of highest prediction coinciding much more closely with the observed range of the species (Figure 5a). This is a clearer test of the ability of the model to reproduce the abiotic responses in the observations used to build the model. As such we must be clear that this is not a test of the transferability of the model to predict new, independent observations (Wenger & Olden, 2012; Yates et al., 2018). Rather it is a validation of the fit of the model to the observations upon which the model was based. The greatest difference between the two methods for introducing background variables is to be expected where a species exhibits multiple optima so that the median values of explanatory variables for the training data are not representative of any of the individual realized peaks in occurrence. *Schoenus nigricans*, a tussock‐forming rush that has distinct ecological loci in base‐rich soligenous mires in the low‐rainfall southeast of Britain and in the lower pH, higher rainfall northwest, is an example (Figure 6). Interestingly the model predicts lower values away from the high‐ and low‐rainfall extremes despite a large number of observations being found in this range (Figure 6a). The model therefore appears to be a poor fit to the observations even though the observations are a reasonable representation of the ecological range of the species in these two dimensions. However, when based on median values for background explanatory variables the pattern is substantially worse (Figure 6b). The highest probabilities all occur outside of the observed ecological range of the species and again the probabilities are lower. Solving the models based on median background variables in the training data is therefore likely to have resulted in an assessment of poorer model fit to either axis than if model predictions were based on observed values at each sample point.

These considerations suggest that there are a number of ways of achieving improved model presentation for assessment. More complex yet information‐rich summaries of the modeled niche are possible to produce but they are likely to take longer to evaluate. Surface plots showing observed presences overlaid with model predictions more clearly show the extent to which the small ensemble of model types has reproduced the observed data. Solving the models using observed values of explanatory variables for each location rather than median values across all locations also avoids applying unrealized and unrealistic combinations of input variables that do not do justice to the fit of the model to observations.

### 3.6 The value of expert elicitation

Human judgment is affected by a range of known biases (McCarthy et al., 2004; Tversky & Kahneman, 1974) and experts are no exception yet their opinions carry greater weight than the nonexpert and therefore have the potential for great benefit if correct (Ellenberg, 2014) or grave disbenefit if false (Hill, 2004). Having two experts assess our niche axes was better than having one. Yet just as the power of the ensemble approach to modeling relies on a consensus among models that reduces the eccentric influence of any one model (Araújo & New, 2006; Smart, Henrys, et al., 2010) it would be desirable to have more experts carry out the model assessment. The size of the task is large however, given the many species and niche axis combinations. A way forward would be to expose the MultiMOVE models to crowd‐sourced expertise. We have implemented this step by presenting bivariate modeled niche surfaces and associated training data in a publicly available online application (https://shiny-apps.ceh.ac.uk/find_your_niche/). Here assessments can now be captured along with a self‐reported indicator of level of expertise. Such an approach allows for more complex yet informative model summaries to be presented since volunteer assessors can take as much or as little time as required for each species of interest. The disadvantage is that no prior control can be exercised over the expertise of the assessor nor the rate at which species models are assessed.

Our results show that statistical and expert assessments of models can be very different for a number of reasons: models can be a poor representation of the phenomena of interest but fit their training data well indicating that the shortcoming is with the observations rather than the modeling method. In addition, simple model summarizes, designed to be readily evaluated by the ecologist but nonexpert in statistics and modeling, can be over‐simplifications. Moreover, experts may have too much faith in the transferability of their own expertise. Our results also confirm the variation that can occur among experts when asked the same question despite their expertise ostensibly covering the same knowledge domain; in this instance the habitat preferences of the British vascular plant flora (e.g. Gastón et al., 2014; Murray et al., 2009; Appendix S1). Having more experts assess the models becomes an obvious requirement when a small number fail to reach consensus. The key lessons from our investigation are (a) that a robust consensus among experts should be based on as large a number of experts as possible, (b) that excessively simple model summaries should be avoided even though this will necessitate additional time for assessment and additional training of experts to interpret more complex model summaries.

## CONFLICT OF INTEREST

None declared.

## AUTHOR CONTRIBUTIONS

SMS designed the study, carried out analysis and led the paper writing. SGJ, MW, SMS and JA programmed the Shiny web application. RHM, CH‐J and TM advised on model validation approaches. ZF and AB computed the AUC values for each model. All authors wrote the paper.

## Data Availability

The MultiMOVE R package is freely available via the Centre for Ecology & Hydrology data catalogue at https://doi.org/10.5285/94ae1a5a-2a28-4315-8d4b-35ae964fc3b9. An online shiny application for submitting assessments of the modeled niche surfaces for British plant species is available at https://shiny-apps.ceh.ac.uk/find_your_niche/. This is best viewed in Chrome.
