# Supplementary material for: Assessment of a large number of empirical plant species niche models by elicitation of knowledge from two national experts
Source: Ecol Evol. 2019 Oct 25;9(22):12858–68. doi: 10.1002/ece3.5766 (PMC6875586; doi:10.1002/ece3.5766)
Supplement: Supplementary file 1 [file ECE3-9-12858-s001.docx]

**Literature survey of studies comparing expert opinion with quantitative species distribution models**

A review of studies was undertaken using Google Scholar. We selected this search engine because we required that the full text of each paper was searched for the specified terms (see <https://libguides.lib.msu.edu/pubmedvsgooglescholar>). This is because model evaluation using expert knowledge may not have been the primary aim of the paper and so not mentioned in title or abstract.

400 papers were reviewed of which 25 were relevant since they involved a comparison of model performance with independent expert evidence where this evidence did not contribute to the construction of each model (Table S1). Seven papers reported that empirical species models outperformed expert opinion, two papers reported little agreement between models and experts and 14 papers reported either useful concordance between experts and models or stated that expert knowledge had a useful role to play in the model validation process. The outcomes of the two remaining papers were unclear. The lowest ratios between numbers of experts and numbers of species applied to assessment of plant models, perhaps not surprisingly. Loiselle et al (2007) used one expert to evaluate 76 plant species and concluded that expert assessment was useful in validating species distribution models. Gastón et al (2014) applied six experts to 54 plant species and found good overall agreement between models and expert opinion although significant variation was apparent among experts. De la Estrella et al (2012) recruited three experts to evaluate 185 plant species but their conclusions did not clearly state the outcome of the expert assessment.

Table S1: List of published research papers where experts independently validated species distribution models. Based on examination of the text of the first 400 papers returned from Google scholar based on the search strings (“species distribution model” or “species niche model”) and (“expert opinion” or “expert judgement”) with these terms occurring anywhere in the text. Google scholar queried between 26 and 28/08/2019.

| **Reference** | **Purpose of study** | **Number**  **of experts** | **Number of**  **ecological units modelled** | **Outcome of expert assessment** |
| --- | --- | --- | --- | --- |
| Aizpurua et al 2015 | To determine the relative merits of habitat suitability models (HSM) versus expert-based survey strategies in detecting populations of a rare species. | 7 | 1 bird species | HSM outperformed both expert-based and general-purpose sampling but expert-based also substantially out-performed the general purpose sampling. |
| Anderson et al 2003 | Evaluated predictive models and used expert assessment as part of the criteria to identify optimal models. | 3 | 3 animal species | Found no consistent association between measures of model performance and expert judgement. |
| Bradley 2013 | Compared habitat suitability models driven by records derived from expert-derived maps versus herbarium collections. | 300 expert-derived distribution maps | 9 invasive plant species | Concluded that expert-driven maps were a far superior basis for modelling invasion risk than herbarium record locations. |
| Chunco et al 2013 | Constructing validated species distribution models for a rare and threatened species. | 36 locations from which local knowledge was sought | 1 animal species | Good agreement between empirical SDM and questionnaire results capturing local knowledge. |
| Clevenger et al 2002 | Compared empirical data-driven versus expert-driven models of Black Bear dispersal. | 2 | 1 animal species | Empirical model performed better because of perceived biases in expert’s knowledge. |
| de la Estrella et al 2012 | Creation of reliable SDM for mapping Legume species richness for conservation planning. | 3 | 185 plant species | The conclusions of the expert comparison were unclear but they highlighted their general importance for future work. |
| Doyon et al 2012 | Comparison of post-disturbance vegetation transition probabilities in northern Canada based on expert knowledge versus a dynamic model. | 7 | 6 forest types. | There was significant agreement among experts. This consensus was greater than the agreement between experts and the dynamic model. Patterns of disagreement were vegetation type specific. |
| Drescher & Perera 2012 | Comparison of empirical data-driven versus expert-driven models of forest succession. | 9 | Forest vegetation types in Ontario | Expert and empirical models showed some similarity but differed in their general structure. Empirical model captured a wider range of observed but lower probability successional pathways. |
| Drolet et al 2015 | Comparing expert opinion versus empirical modelling to predict probability of eradicating aquatic non-native species. | 38 | 143 case studies | Evidence-based models out-performed experts. |
| Fourcade et al 2013 | Compared distributions generated by experts versus species distribution models for a rare and threatened species. | 7 | 1 bird species | Good agreement between the MaxEnt SDM and the expert assessment. |
| Gastón et al 2014 | Compared expert opinion and empirical SDM in selecting forest species for restoration. | 6 | 54 native tree species | Good concordance between experts and models but variation between experts. |
| Germaine et al 2014 | Compared expert-derived and statistical species distribution models. | Not reported | 1 animal species | Concluded that users should have most faith in those domains where experts and independent empirical models agreed. |
| Irvine et al 2009 | Explored whether managers with local knowledge can inform distribution models. | 22 | 1 animal species | Incorporating expert opinion increased the coincidence between modelled and observed occurrences by c.80%. |
| Jiménez-Valverde et al 2010 | Compared empirical niche models with range maps based on expert compilations. | Not reported | 20 species | High correspondence between expert-derived maps and statistical models. |
| Johnson et al 2012 | Case-study to compare SDMs based on expert knowledge or empirical data. Models related moose-vehicle collisions to driving and habitat conditions. | 10 | 1 species | Expert assessments were variable. SDM better at predicting large-scale patterns. Overall, empirical SDM only slightly outperformed expert-based models. |
| Loiselle et al 2007 | Used SDM to explore the assumption that herbarium specimens represent species ranges. Validated models using expert opinion. | 1 | 76 plant species | Expert assessment deemed important in validating SDM. |
| Mainali et al 2015 | Comparing and improving methods for modelling future expansion of an invasive species. | 3 | 1 invasive weed | Expert consensus was high and expert opinion seen as useful in validating and selecting best SDM. |
| Milanesi et al 2017 | Compared expert-derived versus statistical habitat suitability models (HSM) to determine least cost dispersal pathways for landscape genetics. | 4 | 1 animal species | Data-driven HSM out-performed experts’ models. |
| Murray et al 2009 | Tested the usefulness of including expert opinion for prediction of species presence within and beyond the geographical domain of expertise. | 9 | 1 animal species | Experts were poor at reaching consensus outside of their knowledge domain but including locally referenced knowledge was useful. |
| Pearce et al 2001 | Evaluated the added value of including expert opinion in faunal SDM. | 3 | 93 animal species | Expert opinion did not improve models. Fine spatial resolutions patterns were more poorly described by experts than coarse. |
| Reside et al 2019 | Tested the hypothesis that combining expert knowledge combined with an empirical species distribution model would lead to better predictions than each independent approach. | 8 | 17 invertebrate species | Combining expert knowledge and SDM led to better model performance. |
| Seoane et al 2005 | Evaluated the added value of including expert opinion in faunal SDM. | 1 | 10 bird species | Expert opinion offered no improvement in predictive ability. |
| Stevenson-Holt et al 2014 | Compared habitat suitability models (HSM) and expert opinion in defining landscape resistance values for dispersal of the invasive Grey Squirrel. | 5 | 1 animal species | Expert model performed acceptably but the HSM had greater precision at finer resolution. |
| van Zonneveld et al 2014 | Used consensus theory to combine expert knowledge in the evaluation of empirical species distribution models. | 45 | 5 tree species | Lower levels of agreement among experts for models considered poor. Greater consensus for good models. |
| Vasconcelos et al 2012 | Compared empirical species distribution models with existing maps derived from experts. | 755 expert-derived maps | 755 animal species | Good agreement between SDM and maps. |

**References**

Aizpurua, O., Cantú‐Salazar, L., San Martin, G., Biver, G., Brotons, L., Titeux, L. 2015. Reconciling expert judgement and habitat suitability models as tools for guiding sampling of threatened species. Journal of Applied Ecology: 52, 1608-1616.

Bradley, B. 2012. Distribution models of invasive plants over-estimate potential impact. Biological Invasions: 15, 1417-1429.

Chunco, A.J., Phimmachak, S., Sivongxay, N., Stuart, B.L. 2013. Predicting Environmental Suitability for a Rare and Threatened Species (Lao Newt, Laotriton laoensis) Using Validated Species Distribution Models. PLoS ONE 8, e59853. <https://doi.org/10.1371/journal.pone.0059853>

Doyon, F., Sturtevant, B.R., Papaik, M.J., Fall, A., Miranda, B., Kneeshaw, D., Messier, C., Fortin, M-J., James, P.M.A. 2012. In: A.H.Perera et al (eds.), *Expert Knowledge and its Application in Landscape Ecology.* Springer, New York. Pgs 189-210.

Drescher, M., Perera, A.H. 2012. Exploring expert knowledge of forest succession: an assessment of uncertainty and a comparison with empirical data. In: A.H.Perera et al (eds.), *Expert Knowledge and its Application in Landscape Ecology.* Springer, New York. Pgs 173-188.

de la Estrella, M., Mateo, R.G., Wieringa, J.J., Mackinder, B., Muñoz, J. 2012. Legume diversity patterns in West Central Africa: Influence of species biology on distribution models. PLoS ONE 7: e41526. <https://doi.org/10.1371/journal.pone.0041526>

Fourcade, Y., Engler, J.O., Besnard, A.G., Rödder, D., Secondi, J. 2013. Confronting expert-based and modelled distributions for species with uncertain conservation status: A case study from the corncrake (Crex crex). Biol.Cons. 167: 161-171.

Gastón, A., García-Viñas, J.I., Bravo-Fernández, A.J., López-Leiva, C., Oliet, J.A., Roig, S., Serrada, R. 2014. Species distribution models applied to plant species selection in forest restoration: are model predictions comparable to expert opinion? New Forests 45, 641-653.

Germaine, S., Ignizio, D., Keinath, D., Copeland, H. 2014. Predicting occupancy for pygmy rabbits in Wyoming: an independent evaluation of two species distribution models. *Journal of Fish and Wildlife Management* 5:298–314; e1944-687X. doi: 10.3996/022014-JFWM-016

Irvine, R.J., Fiorini, S., Yearley, S., McLeod, J.E., Turner, A., Armstrong, H., White, P.C.L., Van Der Wal, R. 2009. Can managers inform models? Integrating local knowledge into models of red deer habitat use. J.Appl.Ecol. 46: 344-352.

Johnson, C.J., Hurley, M., Rapaport, E., Pullinger, M. 2012. Using expert knowledge effectively: lessons from species distribution models for wildlife conservation and management. In: A.H.Perera et al (eds.), *Expert Knowledge and its Application in Landscape Ecology.* Springer, New York. Pgs 153-171.

Jiménez-Valverde, A., Lira-Noriega, A., Townsend Peterson, A., Soberon, J. 2010. Marshalling existing biodiversity data to evaluate biodiversity status and trends in planning exercises. Ecol.Res. 25: 947–957.

Loiselle, B.A., Jørgensen , P.M., Consiglio , T., Jiménez , I., Blake , J.G., Lohmann, L.G., Montiel, O.M. 2007. Predicting species distributions from herbarium collections: does climate bias in collection sampling influence model outcomes? J.Biogeography: 35, 105-116.

Mainali, K.P., Warren, D.L., Dhileepan, K., McConnachie, A., Strathie, L., Hassan, G., Karki, D., Shrestha, B.B., Parmesan, C. 2015. Projecting future expansion of invasive species: comparing and improving methodologies for species distribution modelling. Global Change Biology: 21, 4464-4480.

Milanesi, P., Holderegger, R., Caniglia, R, Fabbri, E., Galaverni, M., Randi, E. 2017. Expert-based versus habitat-suitability models to develop resistance surfaces in landscape genetics. Oecologia: 183, 67-77. <https://doi.org/10.1007/s00442-016-3751-x>

Murray, J.V., Goldizen, A.W., O’Leary, R.A., McAlpine, C.A., Possingham, H.A., Low Choy, S. 2009. How useful is expert opinion for predicting the distribution of a species within and beyond the region of expertise? A case study using brush‐tailed rock‐wallabies *Petrogale penicillata.* J.Appl.Ecol. 46: 842-851. 2009.

Pearce, J.L., Cherry, K., Drielsma, M., Ferrier, S., Whish, G. 2001. Incorporating expert opinion and fine-scale vegetation mapping into statistical models of faunal distribution. Journal of Applied Ecology 38: 412–424.

Seoane, J., Bustamante, J., Díaz-Delgado, R. 2005. Effect of expert opinion on the predictive ability of environmental models of bird distribution. Cons.Biol. 19: 512-522.

Reside, A.E., Critchell, K., Crayn, D.M., Goosem, M., Goosem, S., Hoskin, C.J., Sydes, T., Vanderduys, E.P., Pressey, R.P. 2019. Beyond the model: expert knowledge improves predictions of species’ fates under climate change. Ecol. Appl. 29: e01624.

Vasconcelos, T.S., Rodríguez, M.Á., Hawkins, B.A. 2012. Species distribution modelling as a macroecological tool: a case study using New World amphibians.

Ecography: 35, 539-548.

van Zonneveld, M., Castañeda, N., Scheldeman, X., van Etten, J., Van Damme, P. 2014. Application of consensus theory to formalize expert evaluations of plant species distribution models. Appl.Veg.Sci. 17, 528-542. 2014.
