# Supplementary material for: Assessment of a large number of empirical plant species niche models by elicitation of knowledge from two national experts
Source: Ecol Evol. 2019 Oct 25;9(22):12858–68. doi: 10.1002/ece3.5766 (PMC6875586; doi:10.1002/ece3.5766)
Supplement: Supplementary file 2 [file ECE3-9-12858-s002.docx]

**S1 Supplementary Note: Expert assessment of MultiMOVE niche models**

Guidance notes distributed to experts

After installation you’ll be presented with a screen that looks like the one below (Fig S1). The idea behind the assessment is to build an independent evidence base for how well each species model represents your expert impression of the niche of the species. The niche of each species is described in terms of seven environmental axes that are all shown together on each species page; four are shown below for *Ajuga reptans*. You should evaluate each of these separately by comparing what the response curve implies about the species’ preference with your experience of the species in British habitats. If unsure because you cannot understand the response or you suspect you do not have enough experience of the species’ preferences throughout its range then don’t hesitate to select ‘Cannot evaluate’. Otherwise select one of the other buttons. It is quite possible that a model could perform well along one niche axis and poorly along another.

If you have any comments about the model fit then please enter these into the comments box. We would hugely value further information but appreciate that there are a lot of species to get through.


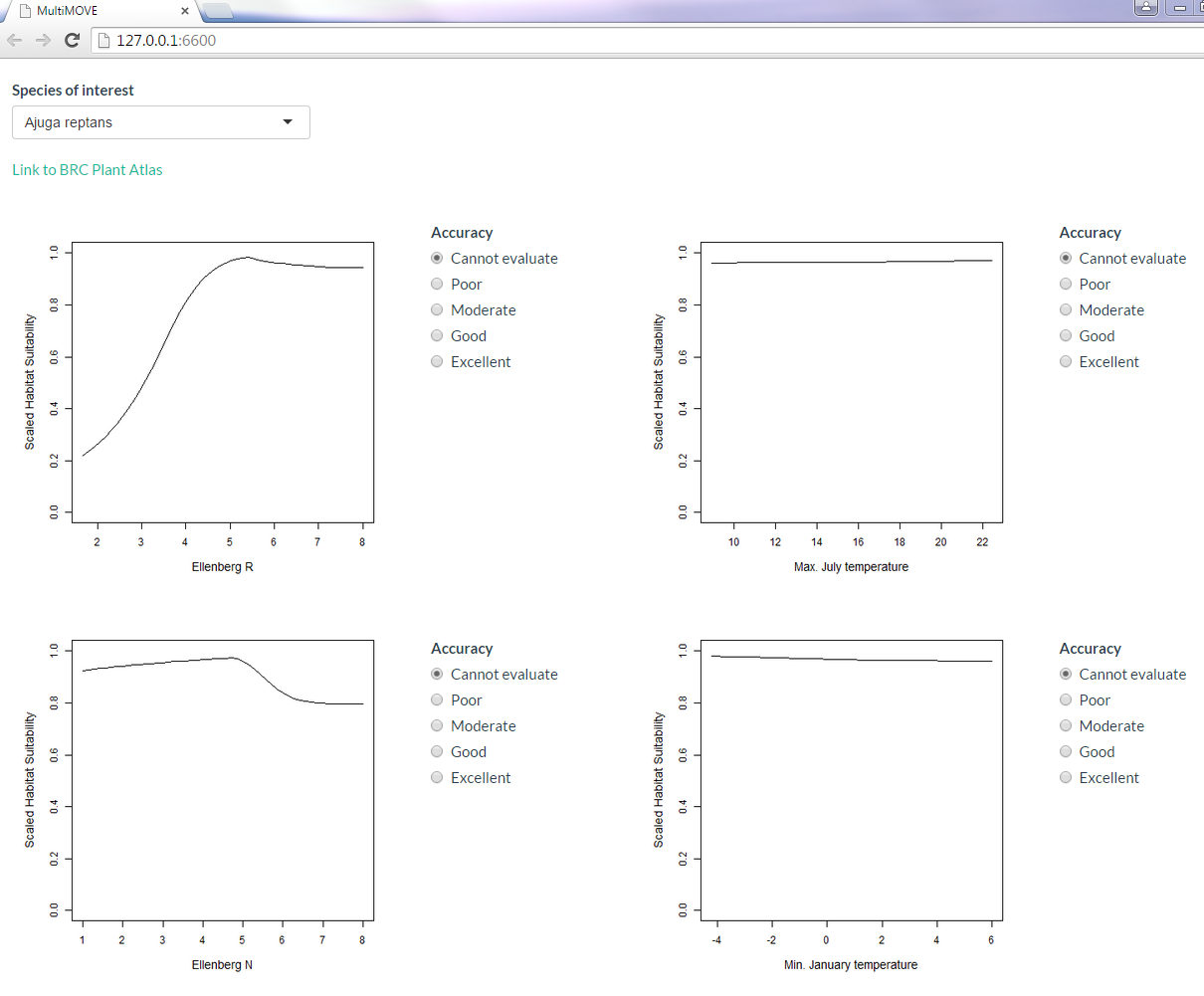


Figure S1.1: Example assessment screen from the Shiny application for expert testing.

In the example above you’ll see that the response of *A.reptans* to two climate gradients – minimum January temperature and maximum July temperature – are essentially depicted by flat lines near to values of 1.0 on the Scaled Habitat suitability axis. This implies that in GB the species is insensitive to these gradients and so does not vary appreciably in its chances of occurrence in warmer versus colder places. You can confirm this for vascular plants by clicking on the ‘Link to BRC Plant Atlas’ link and selecting the hectad map. The one for *A.reptans* is shown below (Fig S2) and indicates that records are spread throughout GB largely consistent with the flat response curves in the model. In this instance you might click anything from Moderate, for example if you were concerned about the lack of records in the extreme north, to Good or even Excellent; it is up to you.


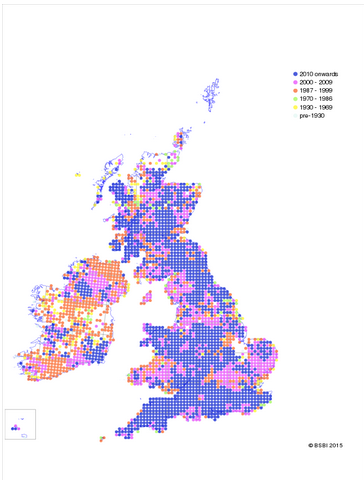


Figure S1.2: Map generated from plant species distribution data collated by the Botanical Society of Britain and Ireland (<http://bsbi.org/>) using the freely available On-line Atlas of the British flora. See <https://www.brc.ac.uk/plantatlas/plant/ajuga-reptans>.

However, there are two important things to note about using the distribution maps in this way.

1. A flat response curve can still be appropriate even if the density of points is much lower as it would be for rare species but where the species’ records are still evenly spread across GB. For rare species you’ll still see that values on the habitat suitability axis will be close to one if the curve is flat. This is because all scores have been rescaled to account for their rarity in Britain. This is so that rare and common can be compared on an equal footing. It just means bearing in mind that high values do not necessarily mean the species is common. They just mean that IF the species were found it would grow happily under the range of conditions conveyed by the way the curve changes along each gradient.
2. Note that a flat response curve could also be appropriate even where there were apparent differences in density across GB. This might occur for example in the *A.reptans* example where an absence of records in the far north could be because of lack of appropriate habitat rather than unfavourable climate space. For example lack of circum-neutral to base-rich deeper soils.

Unfortunately, there is no link to maps for bryophytes nor any links to mapped information on soil conditions. We will investigate whether this is possible or at least send you links to any suitable map data sources that we find. If you know of any then please let us know so we can circulate details.
