# Supplementary material for: Assessment of a large number of empirical plant species niche models by elicitation of knowledge from two national experts
Source: Ecol Evol. 2019 Oct 25;9(22):12858–68. doi: 10.1002/ece3.5766 (PMC6875586; doi:10.1002/ece3.5766)

Fig S2.1 Assessment plot for *Coeloglossum viride*. Simple model average of the probability of occurrence versus the substrate pH axis.


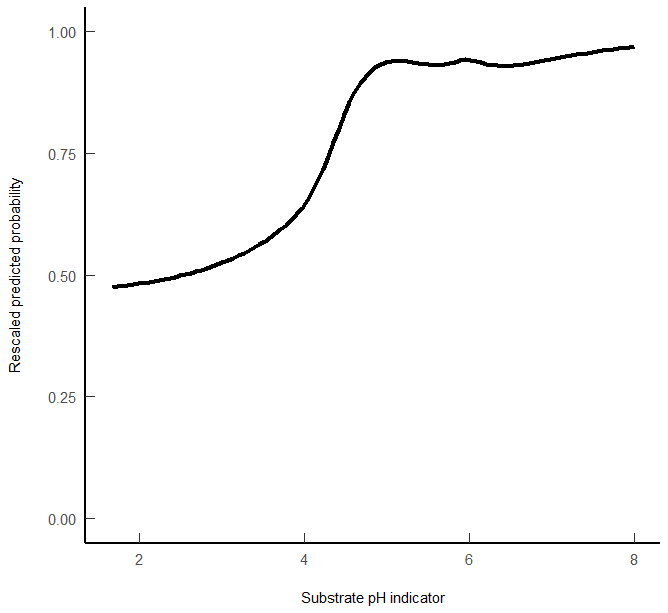


Fig S2.2 Assessment plot for *Coeloglossum viride*. Simple model average of the probability of occurrence versus the vegetation height axis (0, <=10cm; 8, >15m).


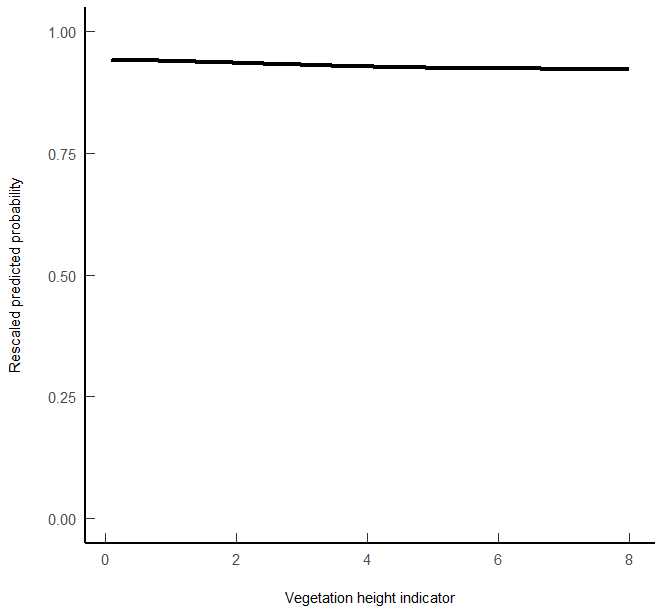
.

Fig S2.3 Assessment plot for *Schoenus nigricans*. Simple model average of the probability of occurrence versus the substrate pH axis.


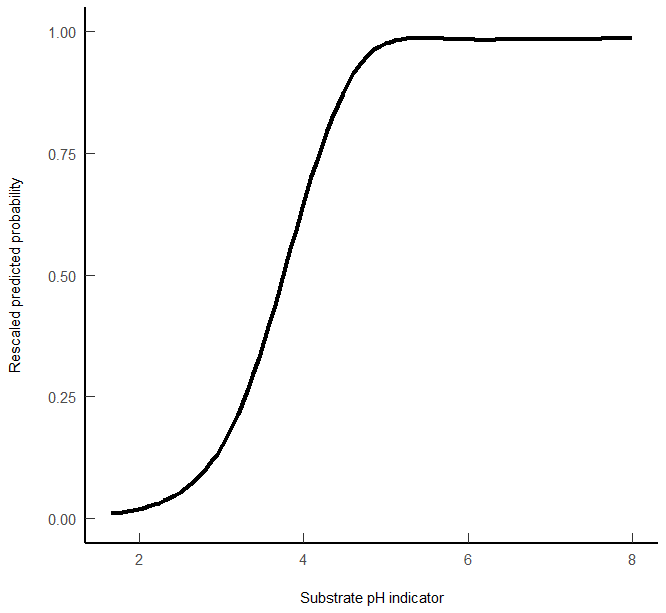


Fig S2.4 Assessment plot for *Schoenus nigricans*. Simple model average of the probability of occurrence versus the annual precipitation axis.


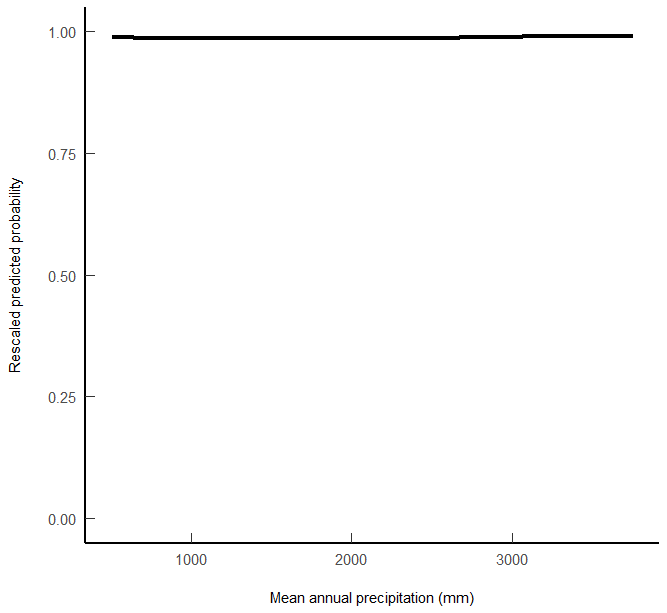

Supplement: Supplementary file 3 [file ECE3-9-12858-s003.docx]
